# A five-m6A regulatory gene signature is a prognostic biomarker in lung adenocarcinoma patients

**DOI:** 10.18632/aging.202761

**Published:** 2021-03-26

**Authors:** Xiao Wu, Hongxu Sheng, Luming Wang, Pinghui Xia, Yiqing Wang, Li Yu, Wang Lv, Jian Hu

**Affiliations:** 1 Department of Thoracic Surgery, First Affiliated Hospital, College of Medicine, Zhejiang University, Hangzhou 310003, China

**Keywords:** lung adenocarcinoma, m6A RNA methylation, prognostic signature, nomogram, survival analysis

## Abstract

We analyzed the prognostic value of N6-methyladenosine (m6A) regulatory genes in lung adenocarcinoma (LADC) and their association with tumor immunity and immunotherapy response. Seventeen of 20 m6A regulatory genes were differentially expressed in LDAC tissue samples from the TCGA and GEO databases. We developed a five-m6A regulatory gene prognostic signature based on univariate and Lasso Cox regression analysis. Western blot analysis confirmed that the five prognostic m6A regulatory proteins were highly expressed in LADC tissues. We constructed a nomogram with five-m6A regulatory gene prognostic risk signature and AJCC stages. ROC curves and calibration curves showed that the nomogram was well calibrated and accurately distinguished high-risk and low-risk LADC patients. Weighted gene co-expression analysis showed significant correlation between prognostic risk signature genes and the turquoise module enriched with cell cycle genes. The high-risk LADC patients showed significantly higher PD-L1 levels, increased tumor mutational burden, and a lower proportion of CD8^+^ T cells in the tumor tissues and improved response to immune checkpoint blockade therapy. These findings show that this five-m6A regulatory gene signature is a prognostic biomarker in LADC and that immune checkpoint blockade is a potential therapeutic option for high-risk LADC patients.

## INTRODUCTION

Lung cancer is a leading cause of cancer-related deaths in the United States of America (USA) with 228,820 newly diagnosed cases and approximately 135,720 deaths expected in 2020 [1, 2]. The most common histological type is lung adenocarcinoma (LADC), which accounts for approximately 49.7% of all lung cancer cases in the USA [3]. In recent years, the incidence rate of LADC has increased significantly [4, 5]. Despite improvements in lung cancer screening and personalized treatment modalities, the 5-year survival rates for stage IV lung cancer patients is ≤10% compared to 68-92% for the stage I patients [6, 7]. Therefore, an accurate prognostic prediction nomogram is required for discriminating high-risk and low-risk patients to determine optimal targeted therapeutic strategies and improve survival outcomes of LADC patients.

N6-methyladenosine (m6A) is the most common and conserved internal transcriptional modification in eukaryotic mRNAs, microRNAs (miRNAs), and long non-coding RNAs (lncRNAs) [8–11]. The m6A RNA modification is highly enriched near the stop codon and 3′ untranslated terminal region (3′UTR), and regulates RNA transcription, variable splicing, translation, and RNA metabolism [12, 13]. The N6 methylation of adenosine is mediated by three categories of m6A regulatory proteins, namely, readers (binding proteins), writers (methyltransferases), and erasers (demethylases) [11, 14, 15]. The readers identify m6A-modified RNAs and include proteins such as IGF2BP1, IGF2BP2, IGF2BP3, YTHDC1, YTHDC2, YTHDF1, YTHDF2, YTHDF3, HNRNPG, HNRNPA2B1, and HNRNPC [16–19]. The writers are methyltransferases that add the methyl group and include proteins such as ZC3H13, RBM15, METTL3, METTL14, METTL16, KIAA1429, and WTAP [18–20]. The erasers are demethylases that remove the methylation group and include proteins such as FTO and ALKBH5 [14, 18, 19, 21]. These three categories of proteins regulate the dynamics of reversible m6A methylation and influence the expression of several genes [11].

The changes in the expression of m6A regulatory genes modulate various physiological processes including self-renewal, circadian rhythm, embryonic development, stem cell differentiation, and cell death [22]. Aberrant m6A modification of RNA is also associated with tumorigenesis [11, 23]. *METTL3* is overexpressed in hepatocellular carcinoma tissues; knockdown of *METTL3* significantly reduced liver cancer cell proliferation, migration, and colony formation *in vitro*, and remarkably suppressed liver cancer tumorigenicity and lung metastasis *in vivo* [24]. Moreover, overexpression of *METTL3* significantly suppresses proliferation, migration, and invasion of colorectal carcinoma (CRC) cells [25]. Therefore, m6A regulatory genes are promising candidates as diagnostic and prognostic markers for various cancers as well as anti-cancer therapy targets [26–28]. Several studies have investigated the role of several m6A regulatory genes in lung cancer [29–34]. However, the role of newly discovered m6A regulatory genes has not been investigated in lung cancer [29–34]. Furthermore, a prognostic risk nomogram including m6A regulatory genes is seldom constructed for lung cancer patients [29–31, 33, 34]. Moreover, association between m6A regulatory genes and tumor immunity has not been explored in lung cancer patients.

Therefore, in this study, we systematically evaluated the prognostic value of m6A regulatory genes in lung adenocarcinoma (LADC) using patient data from the TCGA and GEO databases. Furthermore, we evaluated the association between prognostic m6A regulatory genes and tumor immunity including infiltration status of immune cell types, expression of immune checkpoint proteins, status of tumor mutational burden (TMB), and response to immune checkpoint blockade (ICB) therapy.

## RESULTS

### Seventeen m6A regulatory genes are differentially expressed in lung adenocarcinoma samples

**Figure 1:**
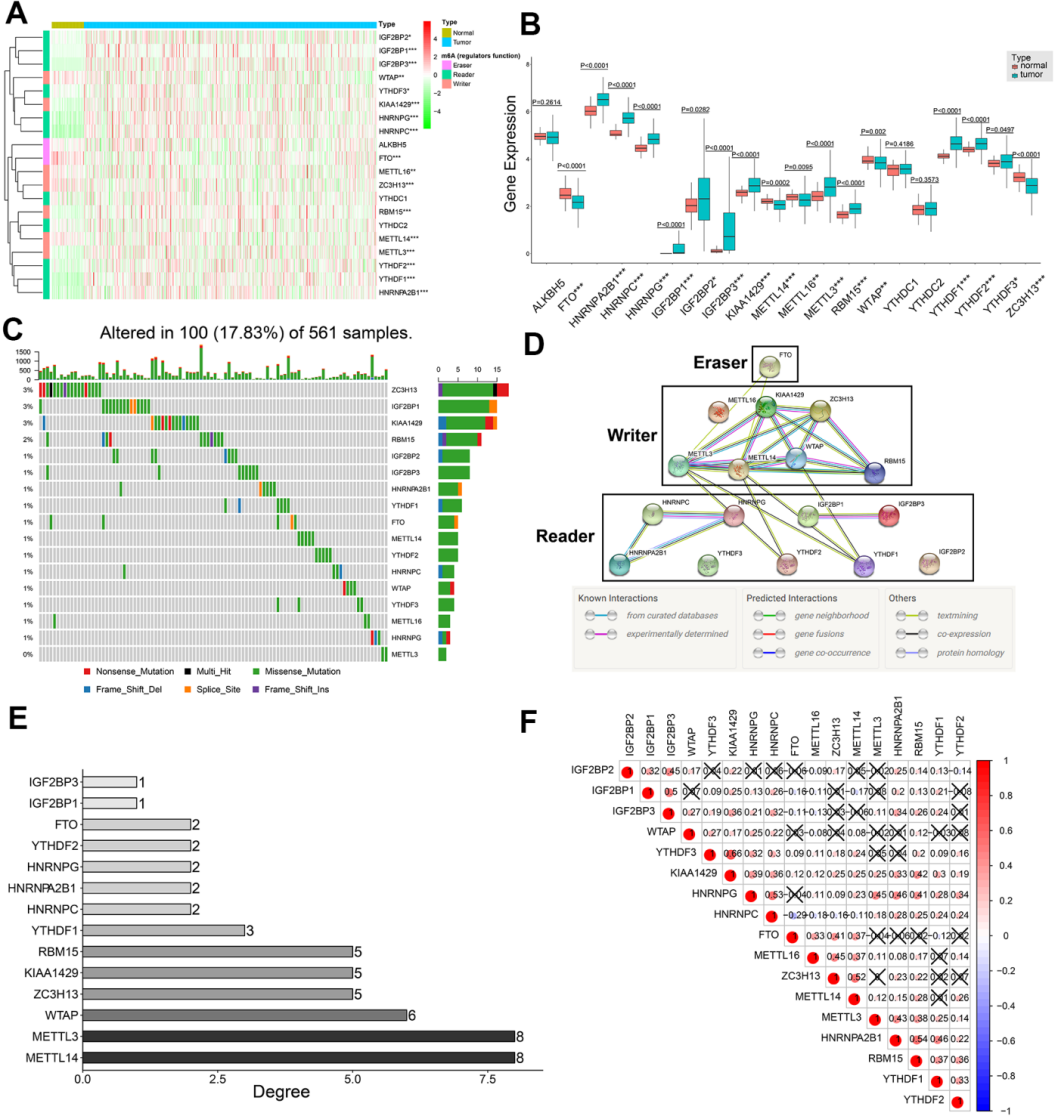
**Expression patterns of twenty m6A regulatory genes in LDAC tissues and protein-protein interaction network analysis of m6A regulatory genes.** (**A**) Heat map shows expression levels of 20 m6A regulatory genes in 59 normal lung and 535 LADC tissues. Note: *0.01 ≤ P < 0.05, **0.001 ≤ P < 0.01, ***P < 0.001. (**B**) Boxplot shows differential expression of the 20 m6A regulatory genes in LADC tissues compared to normal lung tissues. Note: *0.01 ≤ P < 0.05, **0.001 ≤ P < 0.01, ***P < 0.001. (**C**) Single nucleotide variations in seventeen m6A regulatory genes based on analysis of 561 LADC patient tissues. (**D**) PPI network analysis of seventeen m6A regulatory genes using STRING database. (**E**) The interacting numbers of each gene with the other sixteen genes. If the value of one gene is equal to 1, it means that only one of the other sixteen genes was interacted with this gene. (**F**) Spearman correlation analysis between 17 m6A regulatory genes. Blue indicates negative correlation in comparison with red indicating positive correlation. All the correlation coefficients are shown in the squares, and the areas of circles in the squares are positive correlated with the absolute value of corresponding correlation coefficients. Squares containing crosses denotes the P-values of correlation analyses are above 0.05.

We systematically analyzed the transcript levels of twenty m6A regulatory genes in 59 normal lung and 535 LADC tissue samples. The heat map showed that the expression levels of 17 m6A regulatory genes were significantly upregulated or downregulated in LADC tissues compared to normal lung tissues (Figure 1A). *HNRNPA2B1, HNRNPC, HNRNPG, IGF2BP1, IGF2BP2, IGF2BP3, KIAA1429, METTL3, RBM15, YTHDF1, YTHDF2*, and *YTHDF3* were significantly upregulated, whereas, *FTO, METTL14, METTL16, WTAP*, and *ZC3H13* were significantly downregulated in LADC tissues compared to the normal lung tissues (Figure 1A, 1B). However, expression levels of *YTHDC1*, *YTHDC2*, and *ALKBH5* were similar in LADC and normal lung tissue samples (Figure 1A, 1B). We then analyzed single nucleotide variations (SNVs) in the seventeen differentially expressed m6A regulatory genes and found that the mutation frequency was ≤3% (Figure 1C). This suggested that differential expression of the 17 m6A regulatory genes was less likely to be caused by SNVs.

### PPI network and correlation analysis of 17 differentially expressed m6A regulatory genes

Protein-protein interaction (PPI) network analysis of the 17 m6A regulatory genes suggested that *METTL3* and *METTL14* were hub genes (Figure 1D, 1E), whereas, *YTHDF3, IGF2BP2*, and *METTL16* were not linked to any other m6A regulatory genes (Figure 1D, 1E). In general, the ‘writer’ m6A regulatory genes showed significantly higher number of links and the ‘reader’ m6A regulatory genes showed significantly lower number of interacting partners (Figure 1D, 1E). Moreover, all ‘writers’ except *METTL16* interacted with each other, whereas, *METTL16* did not show any interaction with the other writers (Figure 1D). Correlation analysis showed weak to moderate association between the seventeen m6A regulatory genes (Figure 1F). The highest correlation was between *KIAA1429* and *YTHDF3* (r = 0.66), whereas, the correlation between *FTO* and *HNRNPC* was most negative (r = -0.29) (Figure 1F). Besides, *KIAA1429* was the only m6A regulatory gene that significantly correlated with all the other sixteen m6A regulatory genes in the PPI network (Figure 1F).

### Construction of a m6A-gene based prognostic signature

**Figure 2:**
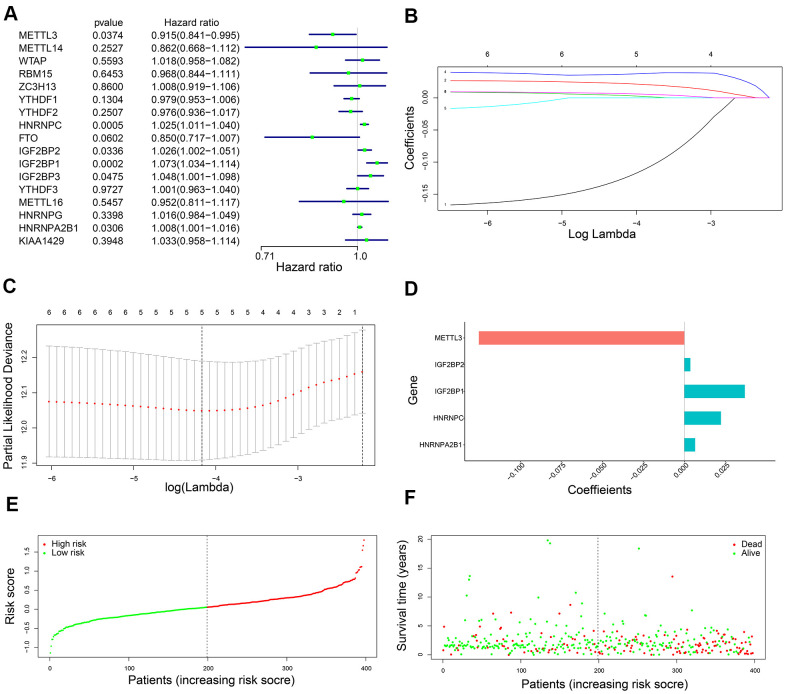
**Development of a five-gene prognostic signature.** (**A**) The forest plots show association between expression levels of seventeen m6A regulatory genes and OS of LADC patients as determined by the univariate Cox regression model. (**B**) LASSO coefficients profiles of the six potentially prognostic m6A regulatory genes. (**C**) The coefficients of five prognostic m6A regulatory genes based on lambda.min using 10-fold cross validation. (**D**) The co-efficient distributions of the five prognostic m6A regulatory genes. (**E**) The distribution of risk scores in the training set LADC patients from the TCGA database. (**F**) The distribution of risk scores of the training set TCGA-LADC patients relative to their OS status.

Univariate Cox regression analysis of the 398 training set LADC patients showed that six out of seventeen m6A regulatory genes, namely, *IGF2BP1, IGF2BP2, IGF2BP3, HNRNPA2B1, METTL3*, and *HNRNPC* were associated with OS (P < 0.05) (Figure 2A). Then, we analyzed these six genes using the LASSO Cox regression model and selected five genes, namely, *IGF2BP1, IGF2BP2, HNRNPA2B1, METTL3*, and *HNRNPC* to construct a five-gene prognostic signature (Figure 2B, 2C). We calculated their coefficients based on the lambda.min via 10-fold cross validation (Figure 2B–2D). The risk score was calculated as 0.037*IGF2BP1 + 0.004*IGF2BP2 + 0.007*HNRNPA2B1 + 0.022*HNRNPC + (-0.125)*METTL3. Then, we classified the training set LADC patients into high-risk and low-risk groups based on the median risk score value of 0.060. We then analyzed the distributions of risk scores and survival status of the training set LADC patients and found that survival times were significantly shorter for the high-risk patients compared to the low-risk group (Figure 2E, 2F).

**Figure 3:**
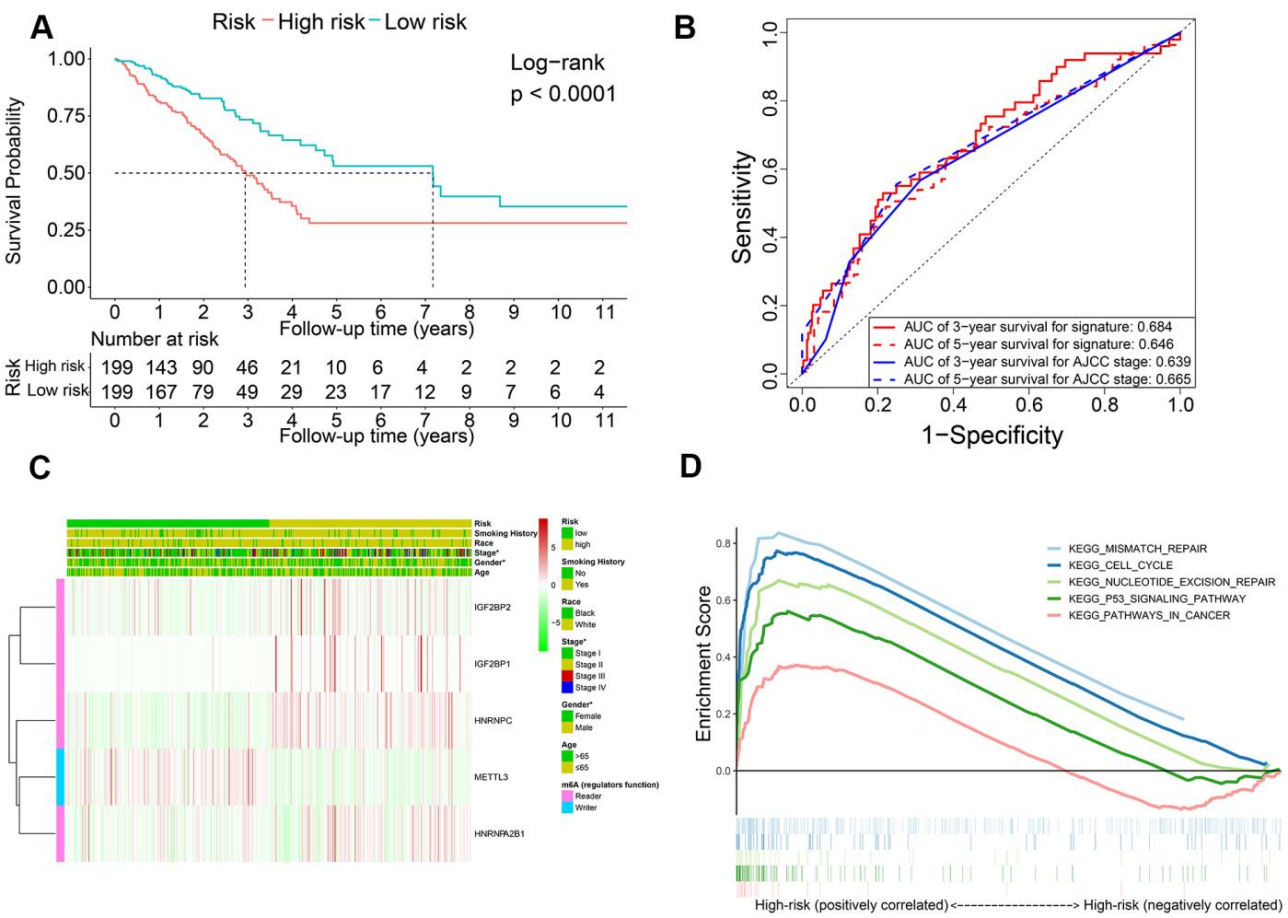
**The association of risk score with OS and clinicopathological characteristics.** (**A**) Kaplan-Meier survival curve analysis of high-risk and low-risk LADC patients from the training set. (**B**) ROC curves show prediction accuracy of the prognostic signature and AJCC stages in the training set TCGA-LADC patients. (**C**) Heat map shows association between the expression levels of the five prognostic m6A regulatory genes and clinicopathological characteristics of high- and low-risk LADC patient subgroups. *0.01 ≤ P < 0.05, **0.001 ≤ P < 0.01, ***P < 0.001. (**D**) The positive correlation between cancer-related pathways and high-risk LADC patients.

Kaplan-Meier survival curve analysis confirmed that OS of the high-risk LADC patients in the training set was significantly shorter than the low-risk LADC patients (P < 0.0001) (Figure 3A). This suggested that the five-gene prognostic risk signature successfully distinguished high-risk and low-risk LADC patients. Furthermore, we performed ROC curve analysis and found that the AUC values of the five-gene prognostic signature for 3-year and 5-year survival of LADC patients were 0.684 and 0.646, respectively (Figure 3B). These AUC values were slightly lower than those considered as acceptable [35]. However, the AUC values for the five-gene prognostic signature were similar to the AUC values for the AJCC stages, which is a commonly used clinical prognostic indicator (Figure 3B). We then analyzed the association between the risk groups and their clinicopathological characteristics. The heat map showed significant differences in gender (P=0.041) and AJCC stages (P=0.028) between the low- and high-risk groups (Figure 3C). We then performed functional enrichment analysis of the differentially expressed genes between the low-risk and high-risk groups. GSEA results showed that the high-risk group was strongly associated with cancer-related pathways including mismatch repair (NES = 2.045, normalized P = 0.004, FDR q-val = 0.005), cell cycle (NES = 2.382, normalized P < 0.0001, FDR q-val < 0.0001), nucleotide excision repair (NES = 2.017, normalized P = 0.006, FDR q-val = 0.006), p53 signaling pathway (NES = 2.069, normalized P < 0.0001, FDR q-val = 0.004), and pathways in cancer (NES = 1.585, normalized P = 0.021, FDR q-val = 0.109) (Figure 3D).

### The five m6A prognostic proteins are overexpressed in LADC samples

**Figure 4:**
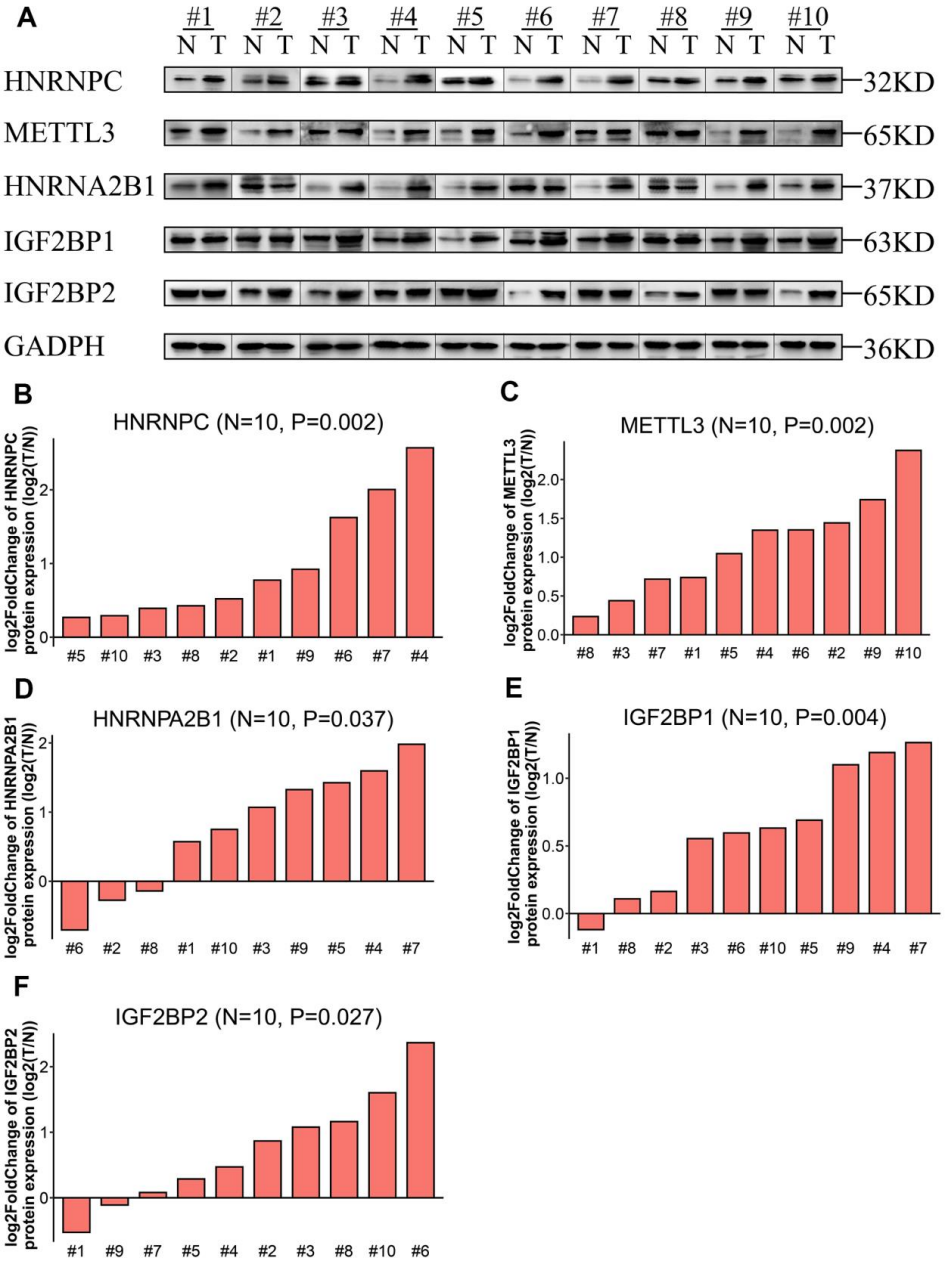
**Expression levels of the five prognostic proteins in LADC patient samples.** (**A**) Western blot analysis shows expression levels of IGF2BP1, IGF2BP2, HNRNPA2B1, HNRNPC, and METTL3 proteins in 10 paired normal lung and LADC tissue samples. (**B**–**F**) Relative expression levels of the five potentially prognostic m6A regulatory proteins, namely, IGF2BP1, IGF2BP2, HNRNPA2B1, HNRNPC, and METTL3 in 10 LADC tissues. GAPDH was used as loading control. The values were normalized by log_2_ fold change (ratio of tumor to normal tissue expression) of the target proteins.

Western blot analysis of ten pairs of matched LADC and adjacent normal lung tissues showed that the expression levels of IGF2BP1, IGF2BP2, HNRNPA2B1, HNRNPC, and METTL3 proteins were significantly upregulated in LADC tissues compared to the corresponding normal lung tissues (Figure 4). This was consistent with the bioinformatics results for the TCGA-LADC dataset.

### Evaluation of the five-m6A regulatory gene signature risk score as an independent prognostic predictor and construction of a predictive nomogram

**Figure 5:**
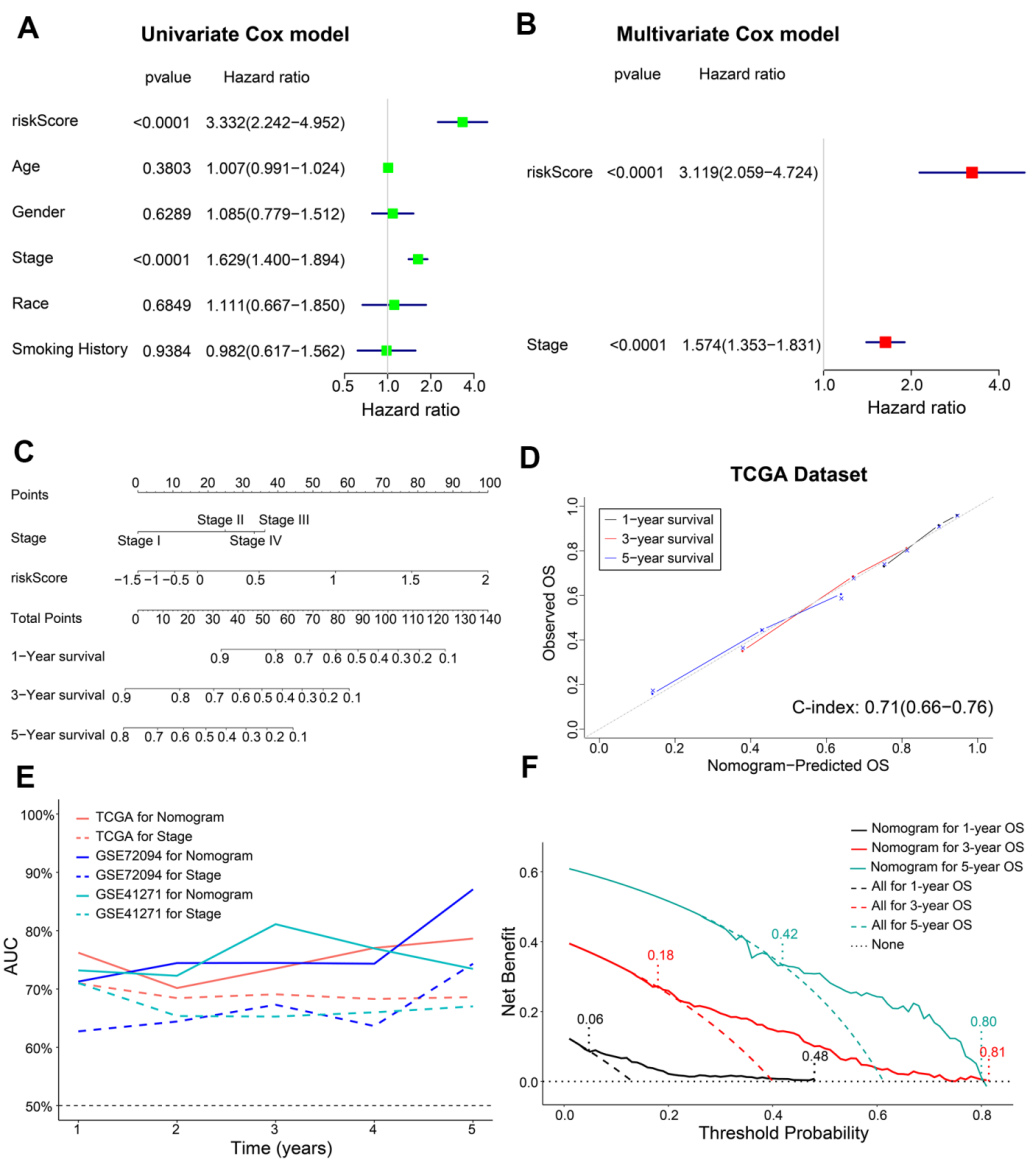
**Construction of a prognostic risk signature-based nomogram and evaluation of its performance in the TCGA dataset.** (**A**, **B**) The forest plot shows association of clinicopathological parameters including risk score and the OS status of TCGA-LADC dataset as assessed by univariate and multivariate Cox regression models. (**C**) The nomogram with risk score and AJCC stages to predict 1-year, 3-year and 5-year OS of individual LADC patients. (**D**) The calibration curves and c-index values for the predicted 1-year, 3-year, and 5-year OS based on the nomogram for the TCGA-LADC patients from the training set. (**E**) ROC curve analysis shows the variations in the AUC values for the nomogram and AJCC stages in the training and validation cohorts for the 1-year to 5-year follow-up period. (**F**) The DCA analysis shows 1-year, 3-year and 5-year OS for LADC patients based on the nomogram.

Next, we performed univariate and multivariate Cox regression analysis to determine if the five-gene signature-derived risk score was an independent prognostic marker using the training cohort (n = 398) LADC patient data. The clinicopathological characteristics of the training cohort are shown in Table 1. Univariate Cox regression model showed that AJCC stage (HR, 1.629; 95% confidence interval (CI), 1.400-1.894; P < 0.0001) and the five-m6A regulatory gene signature risk score (HR, 3.332; 95% CI, 2.242-4.952; P < 0.0001) were prognostic factors for LADC patients (Figure 5A). Furthermore, multivariate Cox regression model also showed that AJCC stage (HR, 1.574; 95% CI, 1.353-1.831; P < 0.0001) and the five m6A regulatory gene signature risk score (HR, 3.119; 95% CI, 2.059-4.724; P < 0.0001) were independent prognostic factors for LADC patients (Figure 5B).

**Table 1:** Clinicopathological characteristics of patients in the training cohort and validation cohort.

**Clinical characteristics**	**Training cohort**		**Validation cohort**	
	**TCGA (No. (% ))**		**GSE72094 (No. (% ))**	**GSE41271 (No. (% ))**
Total	398		320	168
Age at diagnosis				
Median (IQR), years	65 (58-72)		71 (64-77)	64 (57-72)
Gender				
Female	215 (54.0)		170 (53.1)	81 (48.2)
Male	183 (46.0)		150 (46.9)	87 (51.8)
Race				
Black	45 (11.3)		9 (2.8)	11 (6.5)
White	353 (88.7)		311 (97.2)	157 (93.5)
AJCC Stages				
Stage I	219 (55.0)		212 (66.2)	93 (55.4)
Stage II	92 (23.1)		52 (16.2)	27 (16.1)
Stage III	64 (16.1)		46 (14.4)	44 (26.2)
Stage IV	23 (5.8)		10 (3.1)	4 (2.4)
Smoking History				
No	60 (15.1)		30 (9.4)	16 (9.5)
Yes	338 (84.9)		290 (90.6)	152 (90.5)
Survival Status				
Alive	257 (64.6)		233 (72.8)	103 (61.3)
Dead	141 (35.4)		87 (27.2)	65 (38.7)
Survival Time (months)	21.0 (13.9-35.2)		28.1 (19.3-34.4)	39.0 (20.0-64.5)

Abbreviations: IQR: interquartile range; AJCC: American Joint Committee on Cancer.

We then constructed a nomogram based on coefficients from multivariate Cox regression model for the AJCC stage and the five m6A regulatory gene signature risk score and predicted the 1-year, 3-year and 5-year survival probabilities of LADC patients by calculating the total score for each patients by adding the variable values for AJCC stage and risk score (Figure 5C).

Next, we estimated the discrimination efficacy and prediction accuracy of the nomogram in the training set by evaluating c-index and AUC values as well as calibration curves. Our results showed that the nomogram was well calibrated because the curves were close to the diagonal line (Figure 5D). The c-index value for the nomogram was 0.71 (CI, 0.66-0.76) (Figure 5D), and the AUC value was 0.75 in the follow-up period (Figure 5E). Thus, the nomogram showed a higher discrimination power than the conventionally used prognostic index—AJCC stage (Figure 5E). Furthermore, decision curve analysis (DCA) showed that the threshold probabilities for 1-year, 3-year, and 5-year OS based on the nomogram were 0.06-0.48, 0.18-0.81, and 0.42-0.80, respectively (Figure 5F). The net clinical benefit gained from the nomogram was higher than hypothetical treat-all-patients or treat-none scenarios. These data showed that, the nomogram including AJCC stage and risk score was clinically beneficial for predicting 1-year, 3-year, and 5-year OS of LADC patients.

### Validation of the prognostic model in LADC patient datasets from the GEO database

**Figure 6:**
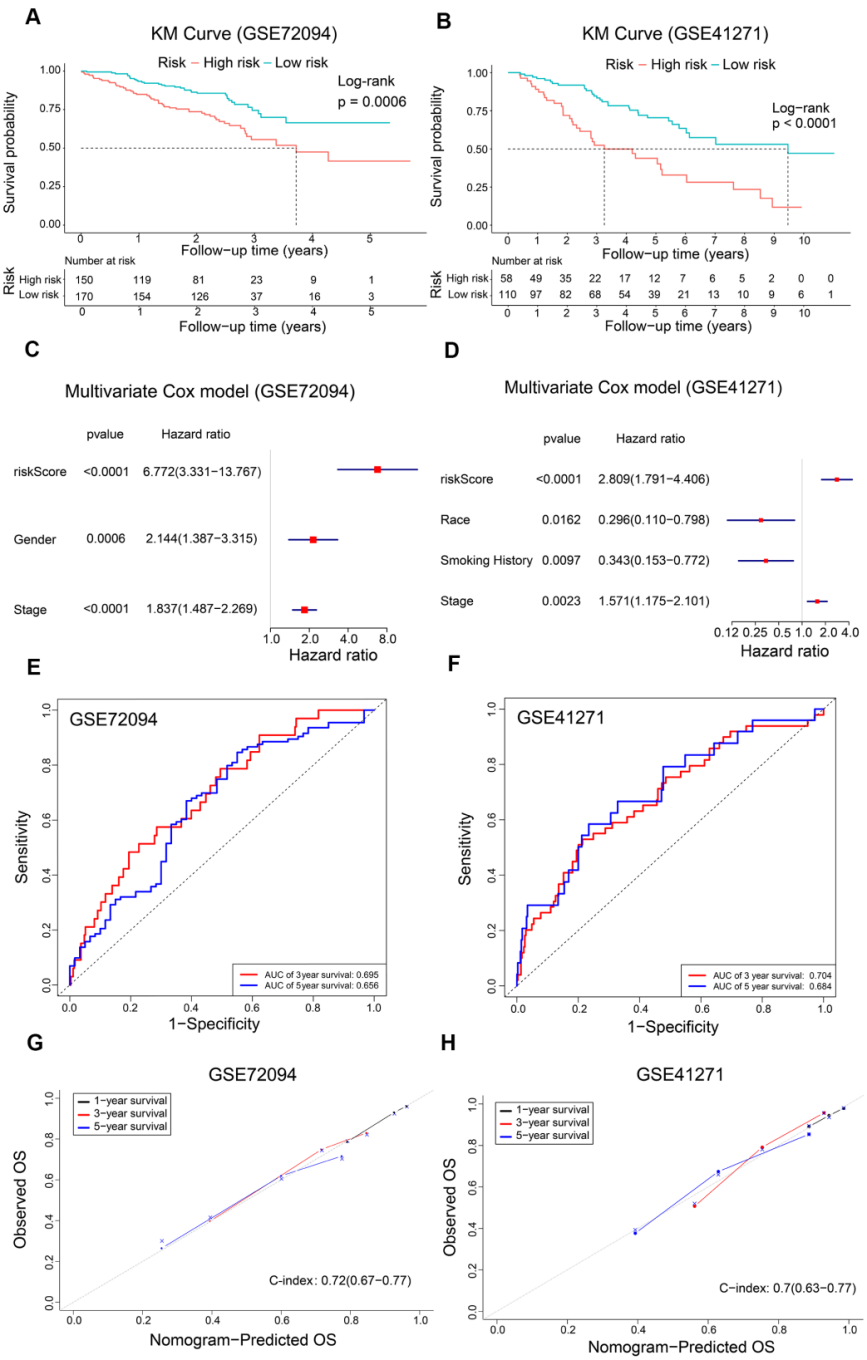
**Validation of the risk signature and nomogram using GEO datasets.** (**A**, **B**) Kaplan-Meier survival curves show OS of high-risk and low-risk LADC patients in the GSE72094 and GSE41271 datasets. (**C**, **D**) The forest plot shows association between clinicopathological characteristics including risk score and OS status as assessed by the multivariate Cox regression model for the GSE72094 and GSE41271 datasets. (**E**, **F**) ROC curve analysis shows the prediction accuracy of the prognostic signature in the GSE72094 and GSE41271 datasets. (**G**, **H**) The calibration curves and c-index values for the predicted 1-year, 3-year, and 5-year OS in the LADC patients from the GSE72094 and GSE41271 datasets based on the nomogram.

We further verified the reliability of the prognostic model using two validation LADC patient cohorts, GSE72094 (n=320) and GSE41271 (n=168). The clinicopathologic characteristics of the validation cohorts are shown in Table 1. We calculated the risk scores of all patients and classified them into high-risk and low-risk groups based on the previously determined cutoff value of 0.060. Kaplan Meier survival curve analyses showed that the OS was significantly shorter for LADC patients in the high-risk group compared to the low-risk group in both GSE72094 (P = 0.0006) and GSE41271 (P < 0.0001) datasets (Figure 6A, 6B). Furthermore, multivariate Cox regression analysis showed that the risk score was an independent prognostic factor for both the GSE72094 (HR, 6.772; 95% CI, 3.331-13.767; P < 0.0001) and GSE41271 (HR, 2.809; 95% CI, 1.791-4.406; P < 0.0001) datasets (Figure 6C, 6D). ROC curve analysis of the prognostic signature showed that the AUC values were 0.695 and 0.656, respectively, for the 3-year and 5-year OS of LADC patients in the GSE72094 dataset and 0.704 and 0.684, respectively, for the 3-year and 5-year OS of LADC patients in the GSE41271 dataset (Figure 6E, 6F). Furthermore, calibration curves for the 1-year, 3-year, and 5-year OS based on the nomogram were close to the diagonal line with a c-index value of 0.72 (CI, 0.67-0.77) for the GSE72094 dataset and 0.70 (CI, 0.63-0.77) for the GSE41271 dataset (Figure 6G, 6H). This suggested satisfactory performance of the prognostic model. The AUC values for both the GSE72094 and GSE41271 datasets were approximately 0.75 for the prognostic model in the 1-year, 3-year and 5-year follow-up period (Figure 5E). These values were higher than the AUC values of approximately 0.65 for the AJCC stage only.

### Identification of co-expression modules and hub genes related to the five prognostic m6A regulatory genes using WGCNA

**Figure 7:**
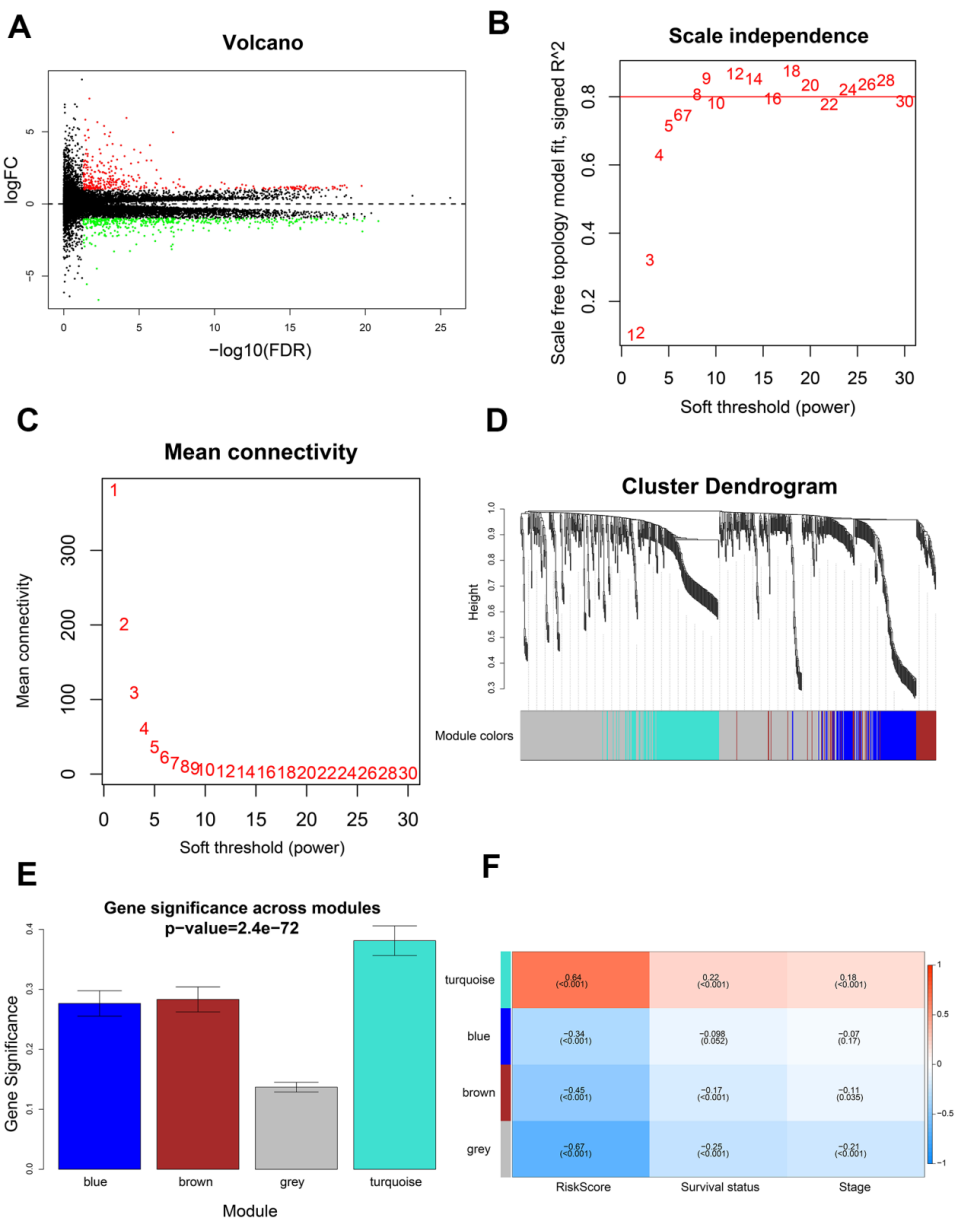
**Identification of gene modules using WGCNA.** (**A**) Volcano plot shows 741 differentially expressed genes or DEGs (335 up-regulated and 406 down-regulated) in 199 high-risk and 199 low-risk LADC patient groups. (**B**) Analysis of scale-free topology fit index for various soft threshold powers (β). (**C**) Analysis of mean connectivity for various soft threshold powers. (**D**) Dendrogram of all differentially expressed genes clustered into various gene modules on the basis of a topological overlap-derived dissimilarity measure. The modules are displayed with different colors in the horizontal bar below the dendrogram. (**E**) Distribution of gene significance values in different modules. (**F**) The module-trait relationship heat map shows the correlation between module eigengenes (MEs) and clinicopathological characteristics. The correlation coefficients and p values are shown in the column for each ME and the corresponding clinicopathological characteristic.

We compared 199 high-risk and 199 low-risk LADC patient groups in the training set and identified 741 differentially expressed genes (DEGs; 335 up-regulated and 406 down-regulated) (Figure 7A). We then developed a co-expression network of the DEGs using WGCNA. We achieved a higher scale-free topology fit index (> 0.8) and higher mean connectivity by constructing a scale-free network with the soft threshold power set as 8 (Figure 7B, 7C). These genes were classified by average linkage hierarchical clustering into three modules, namely, blue (209 genes), brown (173 genes), and turquoise (337 genes) (Figure 7D). We then investigated the association between clinicopathological characteristics and modules by calculating the correlation coefficients between module eigengenes (MEs) and clinicopathological characteristics. The turquoise module showed highest correlation with the prognostic risk score (Figure 7E, 7F).

**Figure 8:**
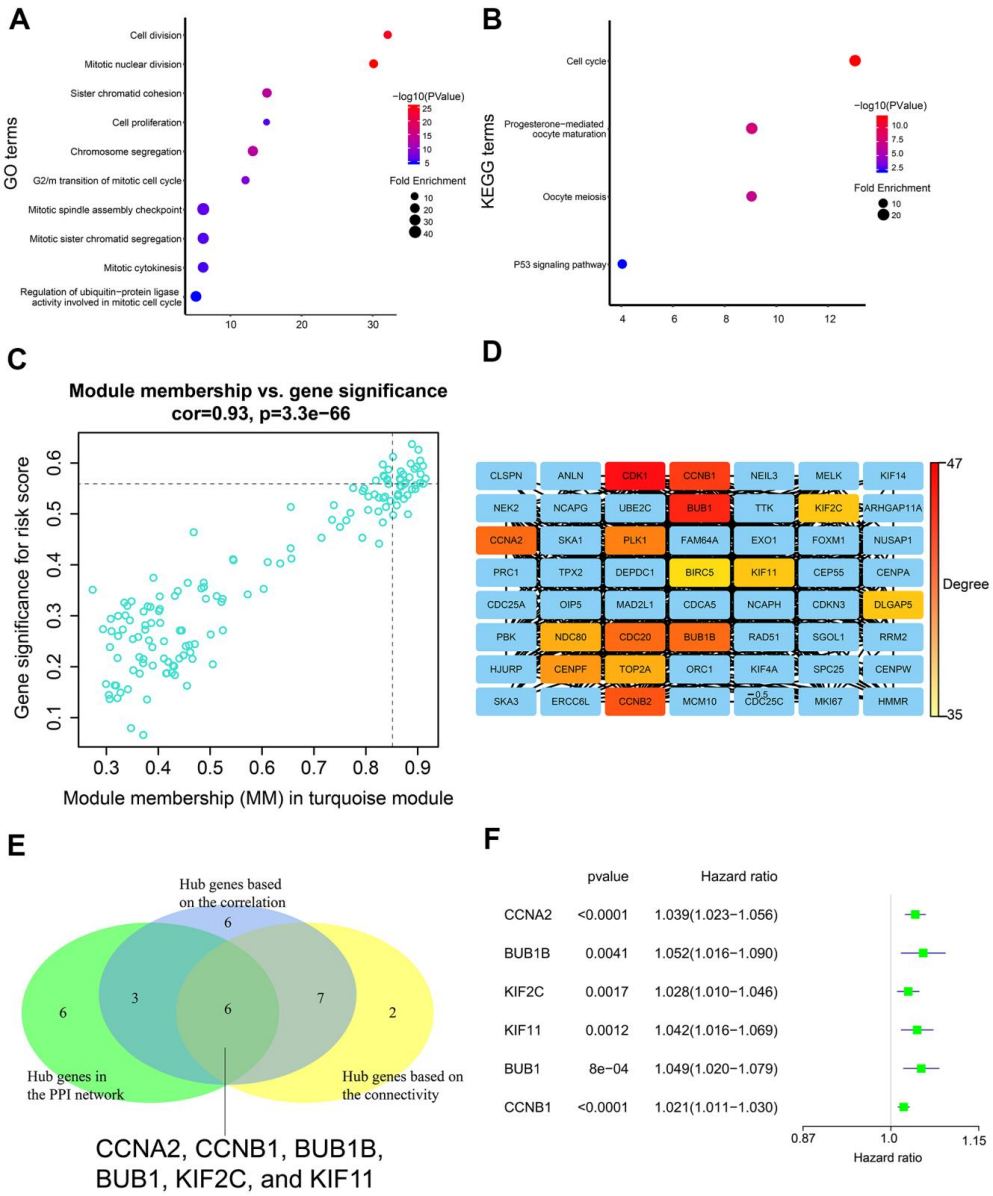
**Gene enrichment analysis in the turquoise module and determination of hub genes in the PPI network.** (**A**) GO enrichment analysis of genes in the turquoise module. (**B**) KEGG pathway enrichment analysis of genes in the turquoise module. (**C**) Correlation analysis of genes in the turquoise module and the risk score. The cutoff values were set as gene significance (GS) > 0.55 and module membership (MM) > 0.85. (**D**) PPI network of all genes in the turquoise module including top 15 hub genes with the highest degree. PPI network was constructed using the STRING database and the Cytoscape software. (**E**) Venn diagram shows six common genes in the three lists of candidate hub genes. (**F**) The forest plot shows association between expression levels of the six hub genes and the OS status of LADC patients as assessed by the univariate Cox regression model.

GO and KEGG enrichment analysis of the turquoise module genes showed enrichment of genes involved in biological processes such as cell division, cell proliferation, and cell cycle, as well as, signaling pathways related to cell cycle, and p53 signaling pathway (Figure 8A, 8B).

We identified 22 genes as potential hub genes because they showed highly significant correlation with the risk score (GS > 0.55) and turquoise module (MM > 0.85) (Figure 8C). Based on connectivity of the weighted network, the top fifteen genes (top 10%) of the turquoise module were designated as the hub gene candidates. Furthermore, we performed PPI analysis of the 337 genes in the turquoise module using STRING database and cytoscape software, and identified the top 15 genes (top 10%) with the highest degree as the hub gene candidates (Figure 8D). By intersecting the three lists of candidate hub genes, we identified six common genes (*CCNA2, CCNB1, BUB1B, BUB1, KIF2C*, and *KIF11*) (Figure 8E). Moreover, the expression levels of these six genes was associated with the overall survival of LADC patients according to the univariate Cox regression model, thereby suggesting that these six hub genes were potential prognostic factors for LADC (Figure 8F).

### Association of m6A regulatory gene prognostic signature with tumor immunity

**Figure 9:**
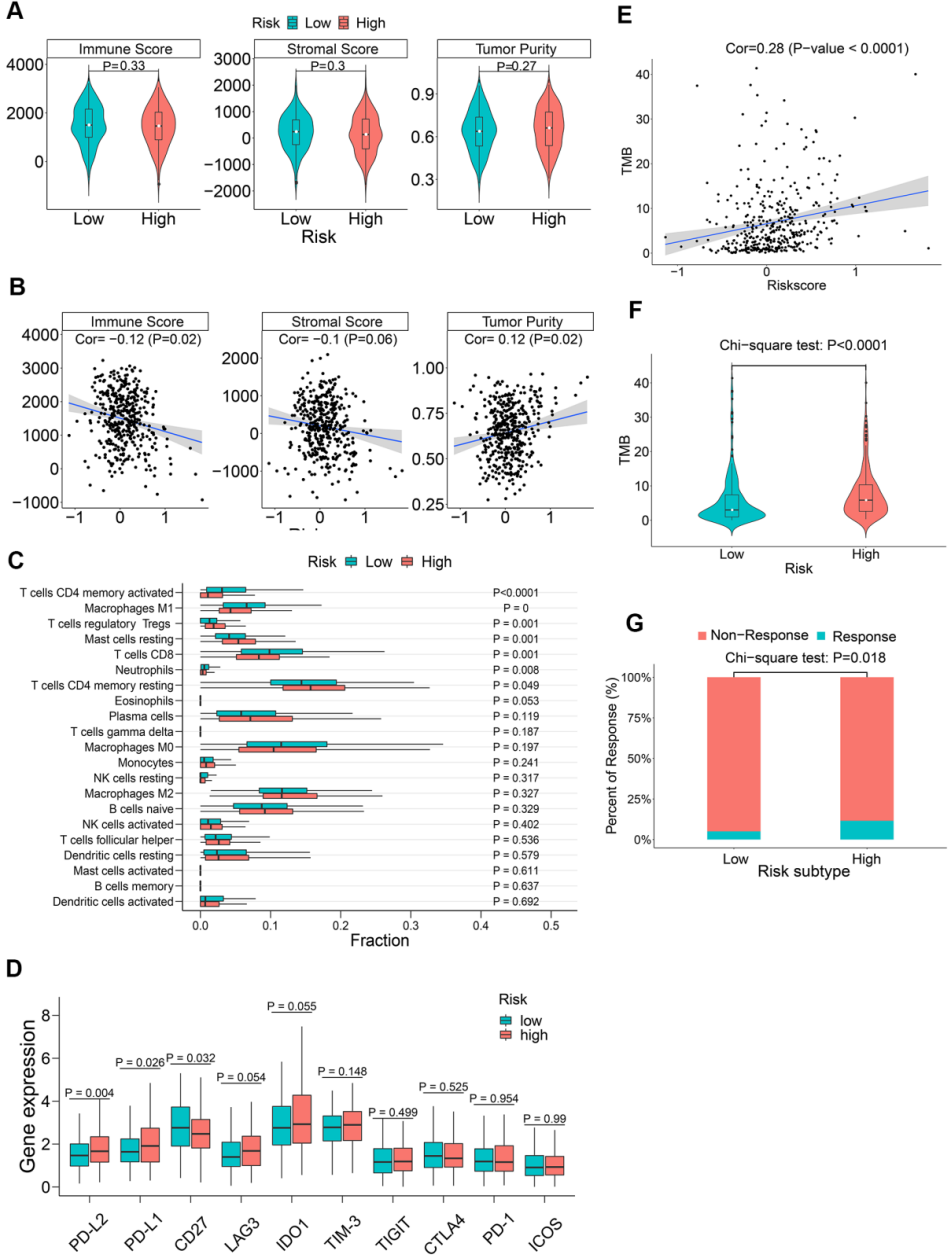
**The association between m6A regulatory gene risk signature and tumor immunity of LADC patients.** (**A**) Boxplot shows differences in immune scores, stromal scores and tumor purity between high-risk and low-risk LADC patient groups. (**B**) Spearman analysis shows the correlation between risk scores and immune microenvironment parameters such as the immune scores, stromal scores and tumor purity. (**C**) Relative proportions of infiltrating immune cell types in the high-risk and low-risk LADC patient groups. (**D**) Boxplot shows differences in expression levels of the immune checkpoint proteins between high-risk and low-risk LADC patient groups. (**E**) Spearman analysis shows correlation between TMB and risk scores. (**F**) Violin plot shows differences in TMB between high-risk and low-risk LADC patient groups. (**G**) Box plot shows the response to immune checkpoint therapy in high-risk and low-risk LADC patient groups.

We used the ESTIMATE algorithm [36] to evaluate tumor immune microenvironment in all LADC patient samples, including immune scores, stromal scores, and tumor purity. In general, we did not observe any significant differences in the immune scores, stromal scores, and tumor purity between the low-risk and high-risk groups (all P > 0.05, Figure 9A). Moreover, the correlation coefficients between the prognostic signature-based risk scores and tumor immune microenvironment scores were all below 0.2 (Figure 9B), which are regarded as negligible [37, 38]. We then investigated the association between the m6A prognostic signature and immune infiltration by evaluating the proportions of 21 immune cell types. The results showed significantly lower proportions of M1 macrophages, activated memory CD4^+^ activated T cells, CD8^+^ T cells, and neutrophils (Figure 9C). We also explored the association between immune checkpoint proteins and the m6A prognostic signature. The results showed significantly higher levels of PD-L1 and PD-L2 in high-risk patients compared to the low-risk patients (Figure 9D). Tumor mutational burden (TMB) is an important biomarker that is useful to predict response to PD-1/PD-L1 targeted immunotherapy in multiple cancer types [39–42]. We observed that the correlation between risk score and TMB was low (r = 0.28) (Figure 9E). Moreover, high-risk patients demonstrated significantly higher TMB (Figure 9F). This suggested that ICB therapy was potentially beneficial for high-risk LADC patients. Finally, in order to assess potential responses of different risk groups to ICB therapy, including CTLA-4 and PD-1 targeted therapy, we performed TIDE algorithm analysis and found that ICB response was significantly higher in the high-risk patients compared to the low-risk patients (Figure 9G). In summary, these findings demonstrate that ICB therapy is potentially beneficial for the high-risk LADC patients.

## DISCUSSION

The prognosis of advanced stage lung adenocarcinoma (LADC) patients is poor [2, 6, 43, 44]. Therefore, a robust prognostic signature that distinguishes high-risk and low-risk patients is essential for determining optimal therapeutic strategies and improving survival outcomes of LADC patients. Previously, studies have investigated the role of thirteen m6A regulatory genes in lung cancer [29–34], but the roles of seven newly discovered ones, namely, *METTL16, YTHDF3, IGF2BP1, IGF2BP2, IGF2BP3, HNRNPG*, and *HNRNPA2B1* has not been determined [29–34]. Furthermore, performance of a robust m6A regulatory gene-based nomogram has not been evaluated with c-index values and calibration curves using training and validation LADC patient cohorts [29–34].

In the present study, we found that seventeen out of twenty m6A regulatory genes were differentially expressed in LADC samples. This suggested their probable role in lung carcinogenesis. Aberrant expression of several m6A regulatory genes is reported in several cancers. *WTAP,* an m6A writer, is up-regulated in hepatocellular carcinoma (HCC) [45], acute myelogenous leukemia (AML) [46], and glioblastoma [47]. Silencing of *ALKBH5*, an m6A eraser, suppresses proliferation and invasion of ovarian cancer cells by enhancing autophagy [48]. *YTHDF1*, an m6A reader, is significantly upregulated in CRC; knockdown of *YTHDF1* significantly inhibits tumorigenicity and colony formation ability of CRC cells [49]. Therefore, dysregulation of m6A regulatory genes is associated with tumor growth, progression, and prognosis of various cancers.

GSEA results showed that m6A regulatory genes were significantly associated with several tumorigenesis-related pathways such as p53 signaling pathway, cell cycle, mismatch repair, and nucleotide excision repair. These results are consistent with previous findings that demonstrate association of m6A regulatory genes with several cancer-related pathways such as p53 signaling pathway [50, 51], cell cycle [50, 52, 53], Ras [52], inflammatory response [50, 51], and PPAR signaling pathway [51].

In the present study, we identified a five-m6A regulatory gene prognostic signature including *IGF2BP1, IGF2BP2, HNRNPA2B1, METTL3*, and *HNRNPC*. This prognostic risk signature accurately predicted the prognosis of LADC patients. Furthermore, we calculated risk scores of LADC patients based on the prognostic signature and stratified LADC patients into low-risk and high-risk groups. Survival analysis demonstrated that the overall survival of high-risk LADC patients was significantly shorter than the low-risk patients.

Previous studies have reported several genetic signature-based nomograms to predict survival outcomes or prognosis of lung adenocarcinoma patients with AUC values between 0.61-0.77 and c-index values between 0.68-0.73 [54–58]. In our study, the AUC value was around 0.75 and c-index value was 0.71 for the nomogram based on the five-m6A regulatory gene based prognostic signature. This demonstrates that the m6A regulatory gene-based risk signature was comparable to previously reported risk signatures based on autophagy-associated genes [54], oxidative phosphorylation-related genes [55], hypoxia-associated genes [56], metabolic-related genes [57], and immune-related genes [58].

Our study identified *IGF2BP1, IGF2BP2, HNRNPA2B1*, and *HNRNPC* as adverse prognostic genes, and *METTL3* as a favorable prognostic gene. These five prognostic genes were highly expressed in LADC tissues. Yan et al*.* demonstrated that *HNRNPC* was significantly up-regulated in non-small cell lung cancer (NSCLC) tissues and promoted proliferation, migration, and invasion of lung cancer cells; high *HNRNPC* expression was also associated with advanced tumor stages, metastasis, and shorter survival time [59]. High expression of *IGF2BP1* promoted proliferation, migration, and invasion of NSCLC cells [60] and liver cancer cells [61]. Zhu et al*.* reported that high expression of *METTL3* was associated with better OS in lung adenocarcinoma patients [24]. Similarly, another study reported that CRC patients with elevated expression of *METTL3* showed significantly better survival than those with lower *METTL3* expression; up-regulation of *METTL3* significantly reduced tumor proliferation, migration and invasion of CRC cells [25, 62]. Our study results are in agreement with these reports. However, other studies have shown contradictory results for *METTL3* in bladder cancer [63], NSCLC [64], and liver cancer [24]. Du et al*.* reported that *METTL3* silencing inhibited the proliferation of lung cancer cells [64]. Another study reported that *METTL3* was significantly up-regulated in bladder cancer, and its knockdown suppressed *in vitro* proliferation, invasion, and survival as well as *in vivo* tumorigenicity of bladder cancer cells [63]. These findings suggest contradictory role of *METTL3* in different cancers [65]. *IGF2BP2*, a direct target of miR-485-5p, is significantly up-regulated in NSCLC, and its depletion significantly suppresses NSCLC cell proliferation and invasion [66]. *IGF2BP2* overexpression is associated with worse OS in pancreatic cancer patients and promotes growth of pancreatic cancer cells by activating the PI3K/Akt signaling pathway [67]. *HNRNPA2B1* is involved in RNA-binding and pre-RNA processing and its high expression is associated with worse prognosis in NSCLC patients; overexpression of *HNRNPA2B1* promotes NSCLC cell growth by activating the COX-2 signaling pathway [68].

We developed a prognostic nomogram based on multivariate Cox regression analysis to predict 1-year, 3-year and 5-year OS probability of LADC patients. The nomogram showed robust prediction performance in both training and validation cohorts. We then performed DCA to ascertain if the nomogram-based clinical decisions could improve patient survival outcomes. The threshold probabilities for 1-year, 3-year, and 5-year OS based on DCA were 0.06-0.48, 0.18-0.81, and 0.42-0.80, respectively, and were more accurate than the hypothetical treat-all-patients or treat-none scenarios. Moreover, the nomogram showed higher predictive accuracy than the traditional prognostic index—AJCC stage. Overall, our findings suggested that the five-m6A regulatory gene signature-based nomogram accurately predicted survival probabilities of all LADC patients and offered a better reference for treatment guidance than AJCC stage alone as the traditional prognostic index.

We then conducted WGCNA to identify the gene modules, hub genes, and signaling pathways associated with the risk signature-related m6A regulatory genes. Cell cycle pathway was significantly associated with the risk signature. We identified six genes (*CCNA2, CCNB1, BUB1B, BUB1, KIF2C*, and *KIF11*) as hub genes, all of which regulated cell cycle progression [69–75]. This suggests that the five m6A regulatory genes (*IGF2BP1, IGF2BP2, HNRNPA2B1, METTL3*, and *HNRNPC*) of the prognostic risk signature regulate cell cycle through the six hub genes. This relationship needs to be experimentally verified in future studies. However, knockdown of FTO, an m6A regulatory gene, inhibits cell cycle progression by increasing m6A levels of *BUB1B and CCNA2* genes, thereby inhibiting their expression [76, 77]. Interaction between IGF2BP1 and KIF11 alters localization of β-actin mRNA, thereby inhibiting cell migration [78]. Several studies have demonstrated that these five m6A regulator genes (*IGF2BP1, IGF2BP2, HNRNPA2B1, METTL3*, and *HNRNPC*) promote cell cycle progression [66, 79–82], but further experimental studies are required to unravel the underlying molecular mechanisms.

Immune checkpoint blockade (ICB) therapy has revolutionized traditional treatment strategies for NSCLC and other cancers. Patients with advanced NSCLC and other cancers demonstrate better prognosis upon treatment with anti-PD-1 and anti-CTLA-4 therapies [83, 84]. Previous studies have also reported that m6A levels play a critical role in immune cell regulation and autoimmune diseases [85, 86]. In this study, we investigated the association of the five-m6A regulatory gene risk signature and tumor immunity. Previous reports suggest that higher PD-L1 expression in tumor cells is closely associated with improved efficacy of immunotherapy [40, 87]. Moreover, stimulation of the PD-1/ PD-L1 pathway promotes apoptosis of CD8^+^ T cells [88, 89]. In this study, we found higher expression levels of PD-L1 and PD-L2, and lower proportions of CD8^+^ T cells in tumor tissues of high-risk LADC patients. This suggested that ICB therapy may be potentially beneficial for the high-risk LADC patients. Moreover, we found higher TMB in high-risk LADC patients. Higher TMB is associated with increased progression-free survival and improved response to PD-1/PD-L1 blockade therapy in multiple cancer types [39–42]. TIDE algorithm analysis demonstrated that ICB response was significantly higher in high-risk LADC patients compared to the low-risk patients. These findings suggest that high-risk LADC patients, as identified by the m6A regulatory gene risk signature, may benefit from ICB therapy.

Our study has several limitations. Firstly, our results were based on data from existing public databases. Therefore, large-scale, prospective, multicenter studies are necessary to further validate our results. Secondly, our study population was mainly white and black patients from the US. Hence, our findings may not be optimal to patients from other countries and races. Thirdly, c-index value of our nomogram was 0.7 and the AUC value of the risk signature was 0.6-0.7. It is plausible that addition of other known prognostic factors such as tumor grade, radiation therapy, chemotherapy, operation modes, and immunotherapy may enhance the prediction accuracy of the present nomogram. Finally, our results were based on data mining and need to be experimentally verified.

In conclusion, we systematically evaluated the expression of 20 m6A regulatory genes in LADC patient tissues and identified a five-gene prognostic signature that can accurately distinguish high-risk and low-risk LADC patients. Furthermore, the nomogram with risk score and AJCC stages accurately distinguished high- and low-risk LADC patients and predicted the survival probability of the LADC patients. We used WGCNA and identified six hub genes that are associated with the cell cycle and related to the five risk signature m6A regulatory genes. We also demonstrated that high-risk LADC patients may benefit from ICB therapy. Further clinical and experimental studies are required to confirm our findings.

## MATERIALS AND METHODS

### Human lung adenocarcinoma tissue samples

We obtained paired LADC and adjacent noncancerous lung tissues from ten patients that were diagnosed through pathological examination at the First Affiliated Hospital of Zhejiang University. We obtained written informed consent from all patients before the tissues were collected. This study was approved by the Ethics Committee of the First Affiliated Hospital of Zhejiang University. The patient samples were frozen in liquid nitrogen and stored at −80° C before further use.

### Human LADC patient datasets

**Figure 10:**
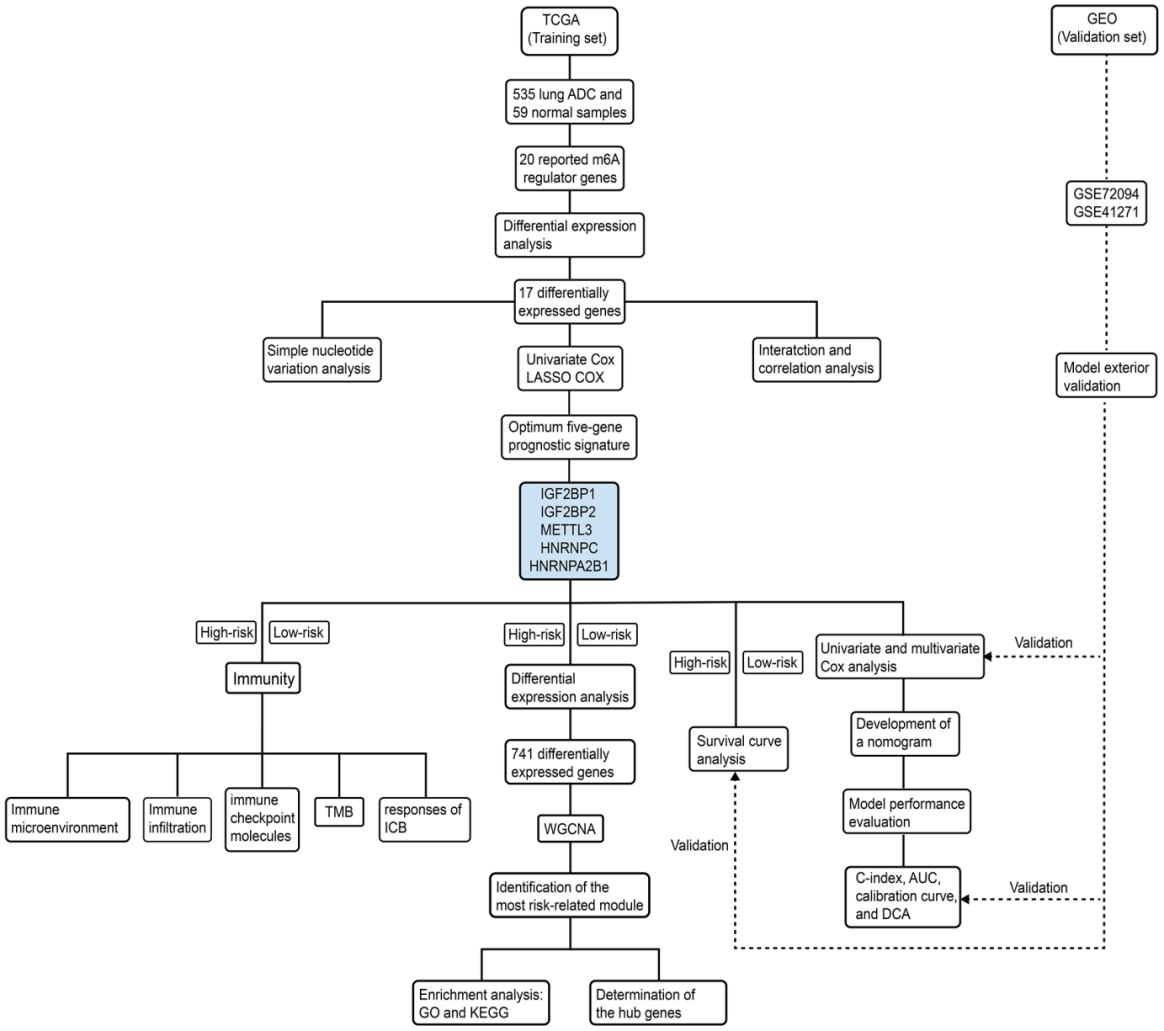
**Schematic representation of study strategy.** The flow diagram shows five main sub-sections of this study—(1) bioinformatics analysis of m6A regulatory gene expression; (2) identification of a five-m6A regulatory gene prognostic signature and construction of a prognostic prediction nomogram; (3) external validation using two GEO patient datasets (GSE72094 and GSE41271); (4) WGCNA, and (5) correlation analysis of the prognostic signature with tumor immunity and immunotherapy response.

The study design and strategy is shown in Figure 10. RNA-seq, clinicopathological, and single nucleotide variation data for 535, 398, and 561 LADC patients were downloaded from the TCGA database (https://portal.gdc.cancer.gov), respectively. RNA-seq data was normalized by the Fragment Per Kilobase Million (FPKM) method to obtain expression values of all genes. We used five LADC datasets from the GEO database (GSE72094, GSE41271, GSE31908, GSE26939, and GSE29013) for validation. Among these, the largest two datasets, namely, GSE72094 and GSE41271 were chosen for independent external validation. The gene expression profiles and clinicopathological data for these datasets were obtained from the Gene Expression Omnibus (GEO) (https://www.ncbi.nlm.nih.gov/geo/) database. Since only a small number of patients were classified as “Asian” or “American Indian/ Alaskan Native”, we focused our analysis on white and black patients.

### Identification of differentially expressed m6A regulatory genes

We systematically compared the mRNA expression levels of twenty m6A regulatory genes, namely, FTO, ALKBH5, ZC3H13, RBM15, METTL3, METTL14, METTL16, KIAA1429, WTAP, YTHDF1, YTHDF2, YTHDF3, YTHDC1, YTHDC2, IGF2BP1, IGF2BP2, IGF2BP3, HNRNPC, HNRNPG, and HNRNPA2B1 [18, 19, 50, 90] in 535 LADC samples and 59 normal lung tissue samples in the TCGA dataset. The differentially expressed genes were determined using the raw p-value corrected for false discovery rate (FDR) and visualized using heat maps and box plots.

### Western blotting

Western blot analysis was performed as described previously [91] using anti-*METTL3* (Cat. no. 15073-1-AP; 1:1,000), anti-*IGF2BP1* (Cat. no. 22803-1-AP; 1:1,000), anti-*IGF2BP2* (Cat. no. 11601-1-AP; 1:1,000), anti-*HNRNPC* (Cat. no. 11760-1-AP; 1:5,000), and anti-*HNRNPA2B1* (Cat. no. 14813-1-AP; 1:2,000) antibodies, all of which were purchased from Proteintech. Anti-GADPH antibody (Affinity; Cat. no. #T0004; 1:3,000) was used to determine GAPDH protein levels. The target protein bands were quantified using the ImageJ v1.53a software (NIH, Bethesda, Maryland, USA). Differential expression was determined by calculating the ratios of target protein values for tumor tissues relative to the corresponding paired adjacent noncancerous lung tissues followed by log_2_FoldChange for each patient.

### Bioinformatics analysis

Gene set enrichment analysis (GSEA) was performed using the GSEA version 3.0 software (Broad Institute, Uc San Diego) to functionally annotate gene sets that show significant differences between high-risk and low-risk LADC patient groups which were classified based on the m6A signature. The cancer-related pathways were extracted based on the following criteria: (1) |normalized enrichment score (NES)|>1; (2) normalized P value < 0.05; (3) FDR q-value < 0.25 [92, 93]. The protein-protein interaction network between m6A regulatory genes was constructed and analyzed using the STRING database (https://string-db.org/).

### Statistical analysis

We compared the expression levels of 20 m6A regulatory genes in 535 LADC samples and 59 normal lung tissue samples using Wilcoxon-Mann-Whitney test. We also used Wilcoxon-Mann-Whitney test to compare expression levels of m6A regulatory genes based on age, gender, race, and smoking history of LADC patients. Kruskal-wallis test was to compare expression levels of m6A regulatory genes based on American Joint Committee on Cancer (AJCC) stages. Spearman correlation analysis was performed to investigate the relationships between different m6A regulatory genes.

Univariate Cox regression model was used to determine prognostic m6A regulatory genes. Least absolute shrinkage and selection operator (LASSO) Cox regression model was used to construct optimal prognostic risk signature. We calculated the lambda value corresponding to minimum mean error (lambda.min) for the five prognostic m6A regulatory genes and evaluated their coefficients using10-fold cross validation. The risk score of all patients in the training and validation cohorts was calculated as the sum of expression level of each gene multiplied by its corresponding coefficient. The patients were dichotomized into low-risk and high-risk groups according to the median value of risk scores. The prediction accuracies of the prognostic risk signature and AJCC stages were assessed by the Receiver operating characteristic (ROC) curve analysis.

Chi-square tests were used to compare the frequency distributions of grouped variables (including age, gender, race, smoking history, and AJCC stage) between the low- and high-risk groups. Kaplan-Meier survival curves and log-rank test were used to compare survival times of low- and high-risk groups. Univariate Cox regression model was used to assess the association between variables including risk score, clinicopathological characteristics and OS in the training set. Multivariate Cox regression model was used to identify variables independently associated with overall survival (P < 0.05). For continuous variables such as the prognostic risk score, the restricted cubic splines (RCS) with three knots located at the empirical quantiles (10%, 50%, and 90 %) were fitted to relax the linearity assumption of the model [94]. Then, forest plots were drawn to better visualize the association between each prognostic variable and OS. A nomogram was constructed to predict the 1-year, 3-year, and 5-year survival probability of LADC patients. Furthermore, prognostic model performance in the training and validation sets was evaluated by concordance index (c-index) values, area under the ROC curve (AUC) values, and calibration curves. A decision curve analysis (DCA) was performed to assess the clinical utility of the nomogram [95]. All statistical analyses were performed using the R statistical software version 3.5.2 (R Foundation, Vienna, Austria). A two-tailed *P<0.05* was considered statistically significant.

### Weighted gene co-expression network analysis

Differentially expressed genes (DEGs) between the high-risk and low-risk groups were screened using false discovery rate (FDR) < 0.05 and |logFoldChange| > 1 as cut-off parameters. A co-expression network of the DEGs was then constructed using WGCNA. A weighted adjacency matrix was constructed based on the power value β, and transformed into a topological overlap matrix (TOM). Subsequently, gene modules were identified by the dynamic shear approach. The most risk-related modules were determined according to module significance (MS) and correlation coefficients between module eigengenes (MEs) and risk scores. Gene Ontology (GO) and Kyoto Encyclopedia of Genes and Genomes (KEGG) enrichment analyses were performed using the DAVID database (https://david.ncifcrf.gov/). The hub genes were determined according to the following criteria: (1) module membership (MM) > 0.85 and gene significance (GS) > 0.55); (2) top ten percent genes based on connectivity of the weighted network; (3) top ten percent genes based on degree of protein-protein interactions (PPI); (4) genes significantly associated with overall survival (P < 0.05).

### Tumor immunity analysis

ESTIMATE algorithm was used to calculate immune score, stromal score, and tumor purity of all LADC patients [36]. CIBERSORT was used to determine proportions of 22 immune cell subtypes in each sample [96] using P-value < 0.05 as the threshold value. Wilcoxon-Mann-Whitney test was used to evaluate the association between m6A regulatory gene prognostic signature and expression levels of ten immune checkpoint proteins, namely, PD-L1, PD-1, PD-L2, CTLA-4, IDO1, LAG3, TIM-3, TIGIT, CD27, and ICOS. TMB was defined as the total number of the mutations in the somatic coding region per million bases. In this study, the mutational frequency of each sample was computed based on the number of variants divided by 38. As the length of exons was 38 million, the number of variants divided by 38 was just equal to the number of variants per million bases (TMB). Tumor Immune Dysfunction and Exclusion (TIDE) algorithm was used to evaluate the potential response to CTLA-4 and PD-1 targeting ICBs in the high- and low-risk patient groups [97].
